# C-Reactive Protein Predicts Further Ischemic Events in Patients With Transient Ischemic Attack or Lacunar Stroke

**DOI:** 10.3389/fimmu.2020.01403

**Published:** 2020-07-07

**Authors:** Manuela Mengozzi, Frances A. Kirkham, Esme E. R. Girdwood, Eva Bunting, Erin Drazich, Jean Timeyin, Pietro Ghezzi, Chakravarthi Rajkumar

**Affiliations:** ^1^Department of Clinical and Experimental Medicine, Brighton and Sussex Medical School, Brighton, United Kingdom; ^2^Brighton and Sussex University Hospitals NHS Trust, Brighton, United Kingdom

**Keywords:** TIA, lacunar stroke, cerebral ischemia, inflammation, oxidative stress, CRP, peroxiredoxin 1, erythropoietin

## Abstract

Patients who have experienced a first cerebral ischemic event are at increased risk of recurrent stroke. There is strong evidence that low-level inflammation as measured by high sensitivity C-reactive protein (hs-CRP) is a predictor of further ischemic events. Other mechanisms implicated in the pathogenesis of stroke may play a role in determining the risk of secondary events, including oxidative stress and the adaptive response to it and activation of neuroprotective pathways by hypoxia, for instance through induction of erythropoietin (EPO). This study investigated the association of the levels of CRP, peroxiredoxin 1 (PRDX1, an indicator of the physiological response to oxidative stress) and EPO (a neuroprotective factor produced in response to hypoxia) with the risk of a second ischemic event. Eighty patients with a diagnosis of lacunar stroke or transient ischemic attack (TIA) were included in the study and a blood sample was collected within 14 days from the initial event. Hs-CRP, PRDX1, and EPO were measured by ELISA. Further ischemic events were recorded with a mean follow-up of 42 months (min 24, max 64). Multivariate analysis showed that only CRP was an independent predictor of further events with an observed risk (OR) of 1.14 (*P* = 0.034, 95% CI 1.01–1.29). No association was observed with the levels of PRDX1 or EPO. A receiver operating curve (ROC) determined a cut-off CRP level of 3.25 μg/ml, with a 46% sensitivity and 81% specificity. Low-level inflammation as detected by hs-CRP is an independent predictor of recurrent cerebrovascular ischemic events.

## Introduction

Stroke is a leading cause of death and long-term disability worldwide (1, 2). Of the total number of prevalent strokes, more than 80% are ischemic (1). Patients who have experienced a cardiovascular event are at higher risk for further events, with a cumulative recurrence rate of ~5% at 1 year (3, 4). Established risk factors include smoking, hypertension (HTN), high body mass index (BMI), atrial fibrillation, diabetes mellitus, atherosclerosis (1, 5). Finding predictive markers for recurrent cardiovascular events could be vital in identifying potential preventive measures.

There is evidence to suggest that stroke is associated with inflammation (6). High sensitivity C-reactive protein (hs-CRP) detects low level inflammation and correlates with cardiovascular risk in the general population (7–10). In a recent meta-analysis, high levels of CRP were associated with ischemic but not haemorrhagic stroke (9). CRP and other inflammatory biomarkers, such as IL-6 and IL-8, are increased after stroke and have been linked to recurrent vascular events (11–16). However, in one study IL-6 (but not CRP) was associated with higher risk of cerebrovascular events (17) and in patients with large-artery atherosclerotic stroke CRP was associated with poor functional disability but not with recurrent vascular events (18).

Oxidative stress has also been implicated in stroke (19). Peroxiredoxins (PRDXs) are intracellular antioxidant enzymes that can be secreted, and their circulating levels are increased under oxidative stress (20). Circulating levels of PRDX1 are elevated in patients following acute stroke (21) and a recent study highlighted PRDX1 as a biomarker of stroke onset early after stroke (22). Secreted PRDXs may then contribute to the inflammatory pathway by activating the production of inflammatory cytokines (23).

Erythropoietin (EPO) is an hypoxia-induced cytokine and has neuroprotective activities in various models of brain injury, including hypoxia and stroke (24, 25). In a clinical trial, EPO administration after ischemic stroke improved clinical recovery and brain damage in patients not receiving thrombolysis (26, 27). In another study, EPO administration did not affect recurrent stroke or mortality but improved long-term neurological outcome (28). A recent meta-analysis confirmed the protective role of exogenously administered EPO in stroke (29). However, the significance of circulating EPO levels as a cardiovascular risk biomarker is not understood. A study reported that an increase in serum EPO levels after ischemic stroke was associated with a favorable outcome (30). However, high serum EPO levels are also associated with impaired prognosis in patients with chronic heart failure (31).

In this study, we investigated the possible significance of biomarkers related to various mechanisms that could increase the risk of a second stroke after recovery from one. We looked in particular at the role of hs-CRP as a biomarker of inflammation, PRDX1 as a marker of oxidative stress and EPO as a potential neuroprotective mediator in predicting further ischemic events in patients who had experienced a transient ischemic attack (TIA) or lacunar stroke.

## Materials and Methods

### Ethics Statement

The Arterial Stiffness In lacunar Stroke and TIA study (ASIST) was an observational prospective cohort study undertaken at the Brighton and Sussex University NHS Trust. The study was approved by the UK National Research Ethics Service (NRES; 14/LO/0189) and was conducted in accordance with Good Clinical Practice Guidelines and with the Declaration of Helsinki. All patients gave written informed consent.

### Clinical Measurements

Patients for the ASIST study each attended a laboratory visit within 14 days of their diagnosed TIA or lacunar stroke. Baseline clinical and demographic information was collected and clinical measures such as blood pressure (BP), BMI etc. were performed as previously described (32). Arterial stiffness was calculated using cfPWV (carotid-femoral pulse wave velocity) measured with Complior®Artech, France, and with the CAVI® Fukuda, Japan (cardio-ankle vascular index) method. Blood samples were taken for measurement of various biomarkers by ELISA assays. Blood was collected in EDTA-containing tubes and plasma was frozen at −80°C until use.

### Participants

Ninety-six patients with a diagnosis of lacunar stroke or TIA confirmed by a stroke physician were recruited to the ASIST study. Of these, 80 patients with plasma samples were evaluated in this study. The follow-up period ranged from 24 to 64 months (mean 42 months). The patients received standard National Institute for Health and Clinical Excellence (NICE) guidance therapy for stroke or TIA depending on type of event and comorbidities.

### ELISAs

High sensitivity C-reactive protein (hs-CRP), erythropoietin (EPO), and peroxiredoxin 1 (PRDX1) were measured in plasma samples with commercially available ELISA kits (hs-CRP: DRG Instruments GmbH, Oxford Biosystems; EPO: R&D Systems, Bio-Techne; PRDX1: Antibodies-online, Caltag Medsystems). PRDX1 was measured only in 40 patients samples selected at random.

### Statistical Analysis

The normality distribution of the data was determined with GraphPad Prism v8.3.0, using the D'Agostino & Pearson test. Normally distributed data were presented as mean ± SD and not normally distributed data as median [interquartile range]. The statistical significance between the groups with and without further cerebrovascular events was assessed with the Mann-Whitney *U*-test for data not normally distributed and with the Student's *t*-test for data normally distributed, both in GraphPad Prism. Prism was also used to create a receiver operating characteristic (ROC) curve, in order to establish a cut-off point for hs-CRP that optimally predicted the occurrence of further events.

Pearson correlation analysis and univariate and multivariate binary logistic regression analyses were carried out using SPSS v25. Odd ratios (ORs) and 95% confidence intervals (95% CI) were determined. Analysis was adjusted for previous stroke, previous TIA, age, sex, smoking status, diabetes, BMI, atrial fibrillation, diagnosis of HTN, average systolic BP, and average diastolic BP. For all analyses, *P* < 0.05 was considered statistically significant.

## Results

This study aimed to investigate whether plasma biomarkers could predict further ischemic events in patients diagnosed with a TIA or lacunar stroke. PRDX1 was measured in 40 patients and hs-CRP and EPO were measured in 80 patients. Of these, two were excluded because they presented hs-CRP >50 μg/ml associated with white blood cell (WBC) count >10,000/ml, a marker of acute bacterial infections (33), leaving 78 patients. In the mean follow-up of 42 months, 24 further cerebrovascular events occurred. Among the 40 patients where PRDX1 was measured, nine further cerebrovascular events occurred.

Table 1 displays the baseline patient characteristics and the univariate analysis of variables associated with further ischemic cerebrovascular events during follow-up. Among the plasma biomarkers measured, only hs-CRP was associated with an increased risk of recurrent TIA/stroke (*P* < 0.05).

**Table 1:** Descriptive analysis of patients and univariate analyses of variables associated with further ischemic events.

		**Further events**		***P*-value**
	**Total, *n =* 78**	**(–), *n =* 54**	**(+), *n =* 24**	
Previous stroke	26.9 (21)	25.9 (14)	29.2 (7)	0.397
Previous TIA	73.1 (57)	74.1 (40)	70.8 (17)	0.648
Age (years)	70.2 ± 11.2	69.5 ± 10.6	71.8 ± 12.7	0.412
Male sex	67.9 (53)	70.4 (38)	62.5 (15)	0.493
Smoking status	65.4 (51)	66.7 (36)	62.5 (15)	0.721
Diabetes	24.4 (19)	16.7 (9)	41.7 (10)	0.021*
BMI (Kg/m^2^)	27.24 ± 4.8	27.1 ± 4.7	27.4 ± 5.2	0.746
Atrial fibrillation	17.9 (14)	20.4 (11)	12.5 (3)	0.408
Diagnosis of HTN	47.4 (37)	42.6 (23)	58.3 (14)	0.202
Average systolic BP	143.0 ± 18.5	142.3 ± 19.0	144.4 ± 17.7	0.650
Average diastolic BP	79.3 [73.3–88.0]	80.0 [75.1–88.1]	77.3 [71.5–87.6]	0.241
Hs-CRP (μg/ml)	1.9 [0.8–3.4]	1.6 [0.7–3.0]	2.8 [1.2–9.9]	0.019*
EPO (mIU/ml)	8.7 [6.3–12.0]	9.1 [6.4–12.6]	8.2 [5.8–11.7]	0.362
PRDX1 (μg/ml)	6.3 ± 1.0^*$*^	6.2 ± 1.0^∧^	6.5 ± 0.8^§^	0.627

Values are given as mean ± SD or median [interquartile range] for continuous data normally or not normally distributed, respectively, and as percentages (n) for categorical data. TIA, transient ischemic attack; BMI, body mass index; HTN, hypertension; BP, blood pressure; hs-CRP, high sensitivity C-reactive protein; EPO, erythropoietin; PRDX1, peroxiredoxin 1. P-value from univariate logistic regression. Values with statistical significance are in bold (*P < 0.05). ^$^n = 40; ^∧^n = 31; ^§^n = 9.

**Figure 1:**
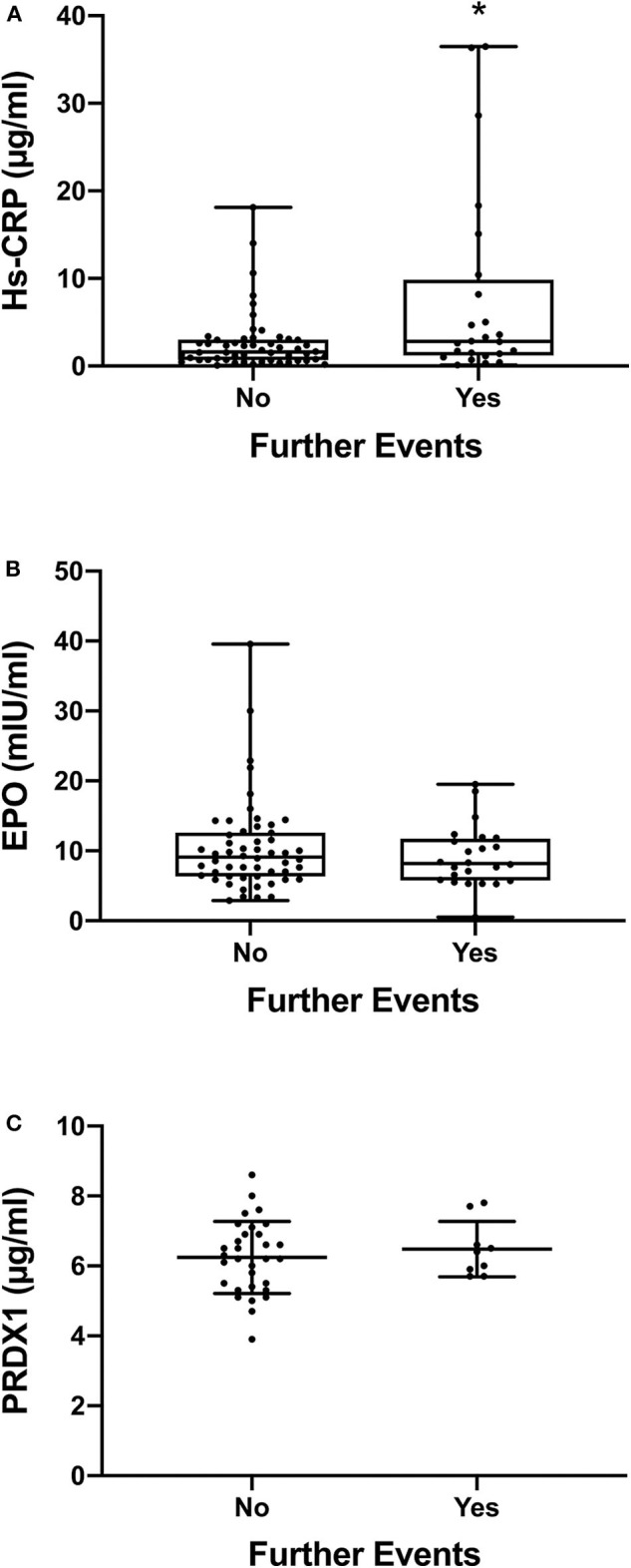
CRP, EPO, and PRDX1 levels in patients. Since the plasma levels of hs-CRP and EPO are not normally distributed, the data in **(A,B)** are presented as box and whiskers plot (minimum, 1st quartile, median, 3rd quartile, maximum); *n* = 54 for the group with no further events and *n* = 24 for the group with further events. The plasma levels of PRDX1 are normally distributed and therefore the data in **(C)** are presented as mean ± SD; *n* = 31 for no further events and *n* = 9 for further events. In all panels, each dot indicates individual data from each subject. *P < 0.05 by Mann-Whitney *U*-test. No significant difference between the two groups of data was detected in **(B,C)** by Mann-Whitney *U*-test and Student's *t*-test, respectively.

Figure 1 shows the levels of the three biomarkers in patients with and without further events. CRP levels (Figure 1A) were significantly higher in the group of patients who experienced a further ischemic event (*P* = 0.041 by Mann-Whitney *U*-test). CRP median levels and interquartile range in the two groups are shown in the figure and also reported in Table 1. There were no significant differences in the levels of EPO (Figure 1B) or PRDX1 (Figure 1C).

We therefore analyzed further the significance of CRP as a predictor of further events. In a multivariate binary logistic regression analysis, after correcting for previous stroke, previous TIA, age, sex, smoking status, diabetes, BMI, atrial fibrillation, diagnosis of HTN, average systolic BP, and average diastolic BP, only CRP was an independent predictor of further cerebrovascular events, with OR 1.14 (*P* = 0.034, 95% CI 1.01–1.29; Table 2). The Pearson correlation between CRP and further events was 0.34 (*P* = 0.002).

**Table 2:** CRP is an independent predictor of further cerebrovascular events.

	**B**	**SE**	***P-*value**	**OR**	**95% CI for OR**	
					**Lower**	**Upper**
Previous stroke	0.214	0.707	0.762	1.238	0.310	4.953
Previous TIA	0.255	0.683	0.709	1.290	0.339	4.917
Age	−0.019	0.039	0.618	0.981	0.909	1.059
Sex	−0.669	0.685	0.329	0.512	0.134	1.963
Smoking status	0.094	0.635	0.882	1.099	0.317	3.813
Diabetes	1.025	0.689	0.137	2.786	0.723	10.742
BMI	−0.024	0.071	0.734	0.976	0.850	1.121
Atrial fibrillation	−0.783	0.912	0.390	0.457	0.076	2.729
Diagnosis of HTN	0.621	0.612	0.310	1.861	0.561	6.178
Average systolic BP	0.030	0.025	0.245	1.030	0.980	1.083
Average diastolic BP	−0.048	0.047	0.311	0.953	0.869	1.046
Hs-CRP (μg/ml)	0.131	0.062	0.034*	1.140	1.010	1.286

Multivariate binary logistic regression analysis. All patients (n = 78) were included in the analysis. B, estimated regression coefficient; SE, standard error; OR, odds ratio. Other abbreviations as in the legend to Table 1. Values reaching statistical significance are in bold (*P < 0.05).

**Figure 2:**
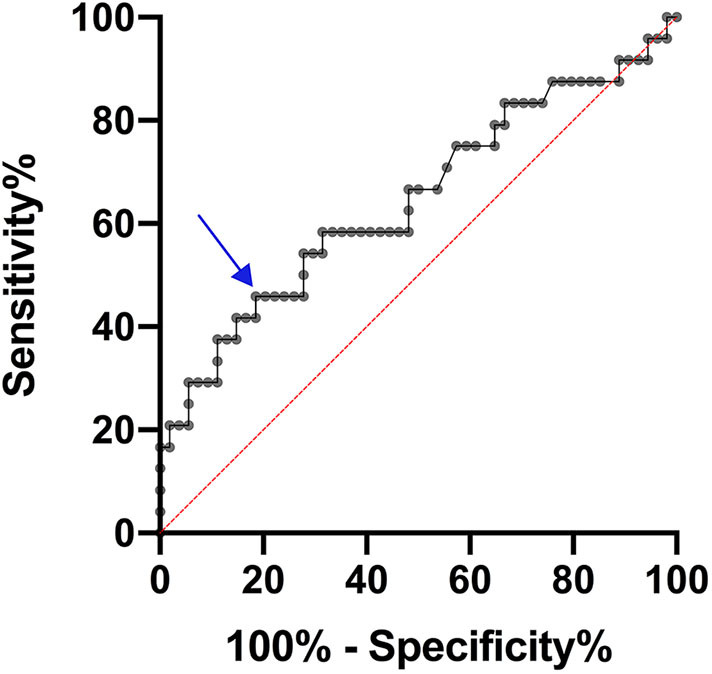
ROC curve for predicting further cerebrovascular events through measure of plasma hs-CRP levels. The arrow shows the cut-off point of hs-CRP = 3.25 μg/ml corresponding to 46% sensitivity and 81% specificity.

We then plotted the levels of CRP as a ROC curve to determine the optimal cut-off value. As shown in Figure 2, a cut-off point of CRP level of 3.25 μg/ml was determined, with a 46% sensitivity and 81% specificity. To further explore the role of CRP in predicting further ischemic events, its levels were assessed as a binary variable using the cut-off value obtained from the ROC curve, and this categorized variable was included in the multivariate analysis (Table 3). This increased the OR from 1.14, obtained using CRP as a continuous variable (Table 2), to 8.32 (*P* = 0.005, 95% CI 1.93–35.91; Table 3).

**Table 3:** Levels of hs-CRP >3.25 μg/ml optimally predict further ischemic events.

	**B**	**SE**	***P-*value**	**OR**	**95% CI for OR**	
					**Lower**	**Upper**
Previous stroke	−0.038	0.743	0.959	0.962	0.224	4.132
Previous TIA	0.322	0.710	0.650	1.380	0.343	5.550
Age	−0.046	0.041	0.264	0.955	0.881	1.035
Sex	−0.832	0.709	0.241	0.435	0.109	1.746
Smoking status	0.279	0.651	0.668	1.322	0.369	4.734
Diabetes	1.335	0.678	0.049*	3.800	1.005	14.361
BMI	−0.039	0.073	0.593	0.962	0.834	1.110
Atrial fibrillation	−0.852	0.887	0.337	0.427	0.075	2.427
Diagnosis of HTN	0.758	0.626	0.226	2.133	0.626	7.275
Average systolic BP	0.035	0.026	0.183	1.035	0.984	1.089
Average diastolic BP	−0.082	0.051	0.103	0.921	0.834	1.017
Hs-CRP >3.25 μg/ml	2.119	0.746	0.005*	8.321	1.928	35.907

Multivariate binary logistic regression analysis including hs-CRP as a categorized binary variable using the cut-off value of 3.25 μg/ml. All patients (n = 78) were included in the analysis. Patients were divided into two groups and hs-CRP was given value 0 in patients with CRP < 3.25 μg/ml (n = 57) and value 1 in patients with CRP >3.25 μg/ml (n = 21). B, estimated regression coefficient; SE, standard error; OR, odds ratio. Other abbreviations as in the legend to Table 1. Values with statistical significance are in bold (*P < 0.05).

## Discussion

This study shows that CRP levels after a minor first cerebrovascular event (TIA or lacunar stroke) can contribute to identifying patients at high risk of a second ischemic event. Elevated levels of CRP (3–6 μg/ml) are a known predictor of ischemic stroke and TIA, as shown in a large study based on the Framingham cohort (7). Subsequent studies have investigated whether CRP levels after an initial ischemic event predict the risk of a second event, in patients with diverse stroke etiologies, measuring CRP at different times after stroke and with different follow-up times.

In general, in patients with major strokes, CRP levels correlate with stroke severity and can be a marker of stroke etiology, with higher CRP in more severe cardioembolic or large artery disease stroke than in stroke caused by small artery disease (13, 34–36). CRP levels increase in the first 48 h after onset, are still elevated at 7 days and remain high for 3–6 months after stroke (11, 34). CRP levels measured after stroke correlated with recurrence both in studies limited to cardioembolic stroke (16) or to large-artery occlusive disease (12) and also in studies where patients with ischemic stroke of different etiologies were included (11, 13). Interestingly, CRP was measured at different times after stroke in different studies, either in the early hours after stroke (11, 13) or between 7 days and 3–6 months from onset (12, 16).

This study was focused on minor stroke, either TIA or lacunar stroke. Since CRP levels are associated with severity and size of the lesion (36), in minor stroke CRP levels are less likely to reflect stroke severity but might be a marker of underlying inflammation and therefore are less subject to variation depending on time from onset. In previous studies in patients with TIA (14) or lacunar stroke (37) CRP levels measured within 24 h or <3 weeks after stroke, respectively, were an independent predictor of further events. Our study, where both patients with TIA or lacunar stroke were included and CRP was measured within 14 days from stroke or TIA onset, confirms the results obtained by Purroy et al. in TIA patients and by Elkind et al. in patients with lacunar stroke (14, 37). Using a ROC curve our study identified a cut-off of CRP of 3.25 μg/ml, measured within 14 days from onset, as an independent predictor of further events. Purroy et al. (14) also used a ROC curve to identify a cut-off of 4.1 μg/ml and Elkind et al. (37) found that higher risk was associated with levels of CRP> 4.85 μg/ml (top quartile). Our results show that even lower levels of CRP (>3.25 μg/ml), measured within 14 days from onset, can help identify patients at risk of second ischemic events after experiencing a minor stroke and support the finding that even low levels of inflammation should be considered a vascular risk factor both in primary prevention and for stroke recurrence (8–10, 37, 38).

Interestingly, in a study focused on TIA patients, Cucchiara et al. found no association between levels of CRP> 3 μg/ml measured within 48 h from onset and risk of second events (39). However, the follow-up in that study was 3 months. In our study the patients were followed up for a longer period of time (mean 42 months, min 24, max 64 months), that was longer than the 1 year follow-up reported by most studies (11–14, 16).

In our study monovariate analysis showed that diabetes is also a predictor of the risk of a further event, in agreement with studies showing that diabetes is associated with recurrent stroke after a first ischemic event (40–42). Higher CRP levels are also known to be associated with the risk of diabetes (43, 44). However, in our study, multivariate analysis showed that CRP is an independent predictor (Table 2), again supporting the view that low-level inflammation is associated with cardiovascular risk.

Our study also explored EPO and PRDX1 levels as potential indicators of the role of hypoxia response and oxidative stress in the occurrence of further events. EPO is typically induced by hypoxia. Activation of hypoxia-inducible factor (HIF)-1α by hypoxia is an adaptive response that leads to the induction of several mediators that can afford protection against hypoxia, including EPO (24). The study by Aberg et al. reported a small increase in circulating EPO in stroke patients compared to healthy controls (mean 9.3 vs. 7.7 mIU/mL, respectively) (30). In that study, higher EPO levels were associated with a more favorable outcome, which would agree with the many preclinical studies on a protective effect of EPO in stroke (25, 45). On the other hand, data from the PREVEND study in patients with heart failure showed that higher EPO levels are associated with an increased risk of stroke in women (46). Our study did not show any association between EPO levels and the risk of further events.

Another transcription factor whose activation may be protective in cerebral ischemia is nuclear factor erythroid 2-related factor 2 (NRF2). NRF2 target genes include several antioxidant enzymes including PRDX1, thioredoxin 1 (TXN1), and heme oxygenase 1 (HMOX1) and its activation affords protection in animal models of stroke (47, 48). Although a previous study reported that the circulating levels of the NRF2 target gene PRDX1 are almost doubled in stroke patients (21), we could not find any significant association in its levels and the risk of further events.

There are some limitations in this study. This is a relatively small study since only 78 patients were included. Blood was taken at the first laboratory visit and therefore within 14 days from onset which was not at the same time from onset for all patients. In addition, PRDX1 was measured in only 40 patients of which nine experienced a further event. The very high variability of PRDX1 levels in patients with no further events, observed after measuring PRDX1 in approximately half of the patients, strongly suggested that PRDX1 was not a good discriminator between the two groups and therefore no further measurements were undertaken; however, we cannot exclude the possibility that significant results could be obtained in a significantly larger study. It is also important to highlight that in our study we did not have a control population; only patients who had an ischemic event were included. Nevertheless, these findings suggest that inflammation, rather than neuroprotective or antioxidant factors (at least EPO and PRDX1), is a possible determinant of the occurrence of secondary ischemic events in patients with TIA or lacunar stroke.

## Data Availability Statement

The raw data supporting the conclusions of this article will be made available by the authors, without undue reservation.

## Ethics Statement

The studies involving human participants were reviewed and approved by UK National Research Ethics Service (NRES; 14/LO/0189). The patients/participants provided their written informed consent to participate in this study.

## Author Contributions

PG and CR designed and supervised the study. MM, EG, and JT did the experimental work. MM, FK, and EG analyzed the data. FK, EB, ED, and JT collected and analyzed clinical data. MM, FK, PG, and CR wrote the manuscript. All authors contributed to the article and approved the submitted version.

## Conflict of Interest

The authors declare that the research was conducted in the absence of any commercial or financial relationships that could be construed as a potential conflict of interest. The reviewer DA declared a past co-authorship with two of the authors MM and PG to the handling editor.
